# Allergens from plant-based food allergen sources and their clinical and diagnostic relevance 

**DOI:** 10.5414/ALX02607E

**Published:** 2026-07-24

**Authors:** Eva-Maria Rick, Melanie Plum, Marua Abu Risha, Uta Jappe

**Affiliations:** 1 Division of Clinical and Molecular Allergology, Priority Research Area Chronic Lung Diseases, Research Center Borstel, Leibniz Lung Center, Airway Research Center North (ARCN), German Center for Lung Research (DZL), Borstel, and; 2 Interdisciplinary Allergy Outpatient Clinic, Department of Pneumology, UKSH and University of Lübeck, Lübeck, Germany

**Keywords:** peanut, legumes, sesame, wheat, tree nuts, fruits

## Abstract

Lately, a rise of plant-related food allergies could be observed following the increase of plant-based food consumption. This narrative review provides an overview of important plant allergens from the most common allergen sources including the “Big 9”, and those gaining more clinical significance such as legumes (other than peanuts) or fruits. Both commercially available and unavailable allergens for in vitro (i.e., specific IgE) and ex vivo (e.g., basophil activation test) diagnostics described in the literature to date are included in this work. In addition, gaps in the commercially available test panels (e.g., oleosins) as well as a lack of knowledge about the clinical relevance of some allergens are highlighted using the latest publications. Furthermore, practical applications are provided in exemplary case reports. In conclusion, molecular allergology provides important tools to advance precision diagnostics of allergic diseases and consequently, patient care.

## Introduction 

Food allergy affects ~ 8% of children and 10% of adults worldwide, though there are significant geographic differences [1]. Primary and secondary food allergy can be discriminated, where the latter is based on IgE cross-reactions between homologous proteins from pollen and food allergen sources (e.g., pathogenesis-related (PR) proteins or profilins) [2]. Food is the most common trigger of severe anaphylactic reactions in children [3, 4]. The most common elicitors for food-based anaphylaxis differ between children and adults: peanut, cow’s milk, hazelnut, cashew, and hen’s egg in children versus shellfish and wheat in adults. However, adults have a broader spectrum of elicitors compared to children [5]. The most common food allergens, the so-called “Big 9”, account for 90% of all food allergies and are comprised of milk, egg, fish, crustacean shellfish, tree nuts, peanuts, wheat, soybean [6] (FDA Food Allergen Labeling and Consumer Protection Act of 2004 (FALCPA)), and sesame, whose inclusion was recommended by the FDA in 2021 (https://www.fda.gov/regulatory-information/search-fda-guidance-documents/guidance-fda-staff-and-interested-parties-evaluating-public-health-importance-food-allergens-other, January 2026). Numerous plant allergen sources such as peanuts, lupine, soybean, tree nuts, sesame, wheat, and celery, among others, have to be declared in bold within the contents list on food packages according to the EU regulation 1169/2011. Within the last two decades, the use of plant-based diets has increased based on healthier life-style choices and ecological concerns [7]. Simultaneously, an increase of plant-based allergies, including less common allergen sources (e.g., peas), has been reported in the literature. 

Diagnosing a food allergy requires a detailed medical history and assessment of sensitization by skin prick testing (SPT) and/or by determining the in vitro specific IgE (sIgE) concentration and/or by oral food challenge (OFC), which is considered the gold standard to determine food allergy [8]. Although not established as a routine diagnostic tool yet, mast cells or basophils stimulated with allergens or allergen extracts (ex vivo tests) can be used to complement the diagnostic procedure (for an overview see [9]). 

In contrast to the SPT and OFC, individual allergens – either purified from food sources or recombinantly produced – can be used to determine sIgE. This component-resolved diagnostics (CRD) is usually more sensitive and specific than tests with whole protein extracts [10]. In contrast to SPTs, a large number of single components can be used in multiplex assays, enabling a large and fast screening process [11, 12]. Scientists and clinicians performing molecular allergy research worldwide still identify new food allergens to date. If submitted to the World Health Organization and International Union of Immunological Societies (WHO/IUIS) Allergen Nomenclature Subcommittee, the allergen is assigned a name based on an abbreviation of the Latin name and a running number after a thorough evaluation process [13], e.g. *Betula verrucosa* (Bet v 1). 

Single allergens can be highly advantageous for detecting food allergy or determining disease severity as presented in a number of publications and case reports in this review. However, only a small fraction of the allergens listed by the WHO/IUIS Allergen Nomenclature Subcommittee is commercially available. For example, oleosins, which are lipophilic proteins absent in aqueous allergen extracts, have often been associated with severe allergic reactions, but are missing in commercial product panels [14, 15]. Another example would be the gibberellin-regulated proteins (GRPs), which are an emerging allergen source within the last decade, and which can cause severe allergic reactions [16]. Allergen components can also help to understand clinically significant IgE cross-reactions, such as the lipid transfer protein (LTP) syndrome [17], or clinically insignificant IgE cross-sensitizations, e.g., between grasses and wheat proteins [18]. Allergen biomarkers or sensitization profiles can even help to discriminate between different disease phenotypes as recently demonstrated in a house dust mite study [19]. These are just a few examples underlining the significance of molecular allergy research. 

This narrative review provides a current overview of plant-based allergens listed by the WHO/IUIS (Table 1, Table 2, Table 3, Table 4, Table 5, Table 6, Table 7, Table 8, Table 9), indicating which components are part of the portfolio from different companies with locations in Germany. The clinical and diagnostic relevance for the most important and/or promising allergens described in the literature are summarized. Applications of molecular allergy diagnostics are shown using exemplary published and unpublished case reports, which underline the significance of commercially unavailable allergens. Proteins from the most common allergen sources are described, namely peanut and its close legume relatives (e.g., soybean, lupine), tree nuts, wheat, and sesame. In addition, lesser known allergens from fruits are presented as they seem to gain more and more clinical significance and should therefore be considered in routine diagnostics. 

## Peanuts and other legumes 

Although a number of different vegetable allergens have been described in the literature (for an overview see [2]) (Tables 1, Table 2), legumes, most prominently peanuts, remain the most significant vegetable allergen sources (reviewed by [20]). Although peanut allergy is well studied, some clinically significant allergens are not commercially available yet. As highlighted in the subsequent sections, many cross-sensitizations exist between peanuts and other legumes, though only some are clinically relevant. 

### Peanuts 

Peanuts (*Arachis hypogaea*) derive from the legume family and are listed among the “Big 9” food allergens. They are one of the most frequent elicitors of anaphylaxis in children [5], and should therefore be avoided when an allergy is known [21]. For a long time, it was recommended that children at risk to develop an allergy based on their family history should avoid peanuts in early life [4]. Some significant studies in recent years have resulted in a paradigm shift that recommends to introduce peanuts into the diet as early as possible to prevent peanut allergy [22, 23, 24]. The OFC is still considered the gold standard to diagnose peanut allergy [25], but some molecular markers can already help in diagnosis: To date, 19 peanut allergens are listed by the WHO/IUIS Allergen Nomenclature Subcommittee (https://www.allergen.org/, January 2026) (Table 3). Ara h 2 [26] is considered the marker allergen for primary peanut allergy in serological diagnostics, demonstrating that CRD can be more sensitive and specific than the use of extracts [27]. It is also associated with severe allergic reactions. The allergenicity of Ara h 1 and 2 increases by roasting in contrast to boiling [28]. Sensitization to Ara h 8, the Bet v 1 homologue in peanuts, is associated with secondary pollen-associated peanut allergy, which mostly triggers mild-to-moderate symptoms [29]. However, it has been previously shown that anaphylaxis can be induced by consuming higher concentrations of Ara h 8 in Bet v 1-sensitized individuals [30]. 

About half of the peanut consists of fat and almost a quarter of proteins (https://fdc.nal.usda.gov/food-details/2515376/nutrients, 20/04/2023). Oleosins [31, 32, 33, 34], along with caleosins and steroleosins [35, 36, 37], are lipophilic proteins that stabilize oil bodies [38, 39], which are storage organelles for lipids in plants, serving as a carbon source and energy storage [39]. The clinical relevance of peanut oleosins has been previously confirmed by Schwager et al. [15], who demonstrated that peanut oleosins can serve as a biomarker for the severity of allergic symptoms. The peanut oleosins Ara h 10, Ara h 11, Ara h 14, and Ara h 15 have been identified to date (Table 3), and the latter was already introduced to a multiplex IgE assay by MadX (Vienna, Austria). Interestingly, oleosins extracted from roasted (processed) peanut seeds have been shown to elicit a stronger immune response from basophils using the basophil activation test (BAT) than those from raw (unprocessed) peanut seeds [15]. During the roasting process, proteins are chemically modified, for example by the Maillard reaction, which helps to create roasting aromas. Despite their strong clinical relevance, oleosins are difficult to implement into routine diagnostics due to their lipophilic nature [14]. 

Since 2020, three additional peanut allergens have been listed by the WHO/IUIS Allergen Nomenclature Subcommittee (www.allergen.org, January 2026), namely Ara h 18 (cyclophilin), Ara h 19 (annexin Gh1), and Ara h 20 (seed biotinylated protein) (Table 3). Ara h 18 does not appear to play a role in the initial sensitization to peanuts, but may lead to positive sIgE test results based on cross-sensitization to homologous allergens in other plants and IgE-binding to cross-reactive carbohydrate determinants (CCDs) [40]. Despite the IgE cross-sensitization with profilin and/or CCDs [40], Ara h 18 has been introduced to the multiplex assay by MadX. In a study with 52 peanut-allergic patients, the prevalence of Ara h 19 sensitization was 46%, but as high as 75% in individuals not sensitized to Ara h 2 [41]. This indicates the potential to detect peanut sensitization in peanut-allergic patients who are serologically negative for the common marker allergen Ara h 2. The same study discovered Ara h 20 sensitization in 10% of patients, which seemed to be moderately linked to symptom severity [41]. Further studies are required to confirm the relationship between Ara h 20 and the severity of allergic reactions to peanuts. 

### Other legumes 

Soybean (*Glycine max*) also belongs to the “Big 9” food allergens, and 0.5% of the European population are affected by soybean allergy [6]. To date, soybean extract and the components Gly m 4 to 6 are commercially available. IgE antibodies to Gly m 4 can cross-react with PR-10 homologues from other plants such as birch (Bet v 1). The prevalence of soybean allergy in patients with birch allergy varies between studies. Mittag et al. [42] found out that only 9.6% of Bet v 1-sensitized patients with birch pollen allergy reported symptoms of soy allergy, whereas others demonstrated soybean allergy in approximately 60% of birch-allergic patients [43, 44]. These variations could be explained by the respective study designs. Based on the data from Husslik et al. [43] and Berneder et al. [45], 70% of patients with birch pollen allergy that also react to soy suffer from severe symptoms after consumption of certain soy-based products. The highest risk of anaphylactic reactions in birch-allergic patients is associated with soybean-based drinks, such as soy milk, milk shakes, and ice cream [46]. Gly m 4 seems to be one of the major triggers for these severe reactions [42, 46, 47, 48]. Although some studies suggest that patients with severe reactions tend to have higher Gly m 4-specific IgE concentrations [46, 47, 48], other studies could not corroborate these observations [43, 49]. The BAT using Gly m 4 can be a useful tool for birch-allergic patients with anaphylactic reactions to soybean as shown by a case study from Evrard et al. [50], however, the PR-10 protein may not be the only trigger of anaphylaxis in these patients [51]. 

The quantity of Gly m 4 differs between food products with the highest levels detected, e.g., in soy milk, which is only briefly heated for pasteurization [42]. PR-10 proteins are considered heat labile [52], however, the grade of Gly m 4 denaturation depends on the heating time and temperature [42, 53]. In addition, Finkina et al. [53] found out that heat-denatured Gly m 4 has the ability to refold and subsequently trigger an IgE response with and without Bet v 1-related epitopes. This allergen is also able to cross the intestinal epithelial barrier, particularly if the gastric pH is not low enough for proper digestion [53]. Therefore, products containing too much undenatured soy protein like soybean-based drinks can pose a risk for patients with birch allergy. 

Albeit lacking clinical relevance in most cases, significant cross-sensitizations between soybean and peanut allergens have been observed [54], for example between Gly m 6 and Ara h 3, Gly m 5 and Ara h 1, as well as Gly m 8 and Ara h 2 [55, 56]. The storage proteins Gly m 5 and 6 in particular have been described as markers of severe allergic reactions to soy products in both children and adults [57, 58]. The heat-stable seed biotinylated protein Gly m 7 might be a promising food allergen for diagnostics as it performed better in the BAT than Gly m 5 and soybean extract [59]. Interestingly, IgE binding was only partially inhibited by peanut extract [59], which indicates the potential for some degree of cross-reactivity between these legumes. The homologous protein/s has/have not been determined yet. 

Over the past 20 years or so, there has been an increased trend towards vegetarian or vegan food in Europe so that legumes other than soybean, such as peas, lentils, lupines, chickpeas, or fenugreek, became a more prominent protein source in our diet. Recently, cases of allergies to these legumes have been more frequently reported, and some relevant allergens have been detected and subsequently registered by the WHO/IUIS Allergen Nomenclature Subcommittee (www.allergen.org, January 2026) (Table 4) (reviewed by [60]). However, current diagnostic approaches rely mostly on legume protein extracts. Therefore, genuine allergic reactions might be hard to detect as cross-sensitizations among legumes are quite common as demonstrated by Muller et al. [61]: 195 peanut-allergic children demonstrated a co-sensitization between lupine, fenugreek, soybean, and lentils in descending order. At least one additional allergy to either lentil, lupine, or pea could be diagnosed in 27.9% of the children. Similarly, a retrospective study [62] using a cohort of 317 peanut-allergic children observed cross-reactions mostly with pea, followed by lentil, soybean, sweet lupine, and chickpea. Although > 60% of children showed a cross-sensitization to bean, this sensitization was not clinically significant. In fact, only 7.9% of all the cross-reactions observed were clinically relevant. However, the current diagnostic measures using extracts are insufficient to identify these cases. 

Lupines (*Lupinus* species) are allergen sources frequently present in different food products such as baked goods and meat-substitution products. Recently, our group successfully identified and isolated LTPs from *L. angustifolius* and *L. luteus* [63], which were able to trigger a positive basophil activation using the blood from a patient with LTP syndrome in our optimized BAT [64]. This highlights that the availability of single legume allergens is of some importance even for people who are not on a vegetarian/vegan diet. This is also relevant for peanut-allergic individuals with cross-reactions to lupines. De novo sensitizations to lupine have been described with almost comparable frequency to cross-reactions with peanuts [65]. 

An allergy to fenugreek was described in cases of both occupational asthma [66] and food allergy [67], where skin reactions may also occur. Consumed in the form of spices (e.g., curry), in cheese, baked goods, confectionery, and possibly also in coffee substitutes and herbal teas, fenugreek may induce allergy in patients with pre-existing peanut allergy. In fact, so far, only 1 case of a primary fenugreek allergy has been suspected [67]. 

Peas are frequently used as an ingredient in meat-substitution products, which poses a risk for pea-allergic individuals. The increased number of published studies [60] infer that the incidence of pea-allergic individuals is on the rise with Pis s 1 described as a major allergen [68]. 

These studies show that cross-reactions amongst legumes are usually of less clinical relevance than suspected. However, they also emphasize the need for single legume allergens with biomarker potential in routine allergy diagnostic tests. The increasing incidences of legume allergy cases strongly implicate the need for accurate and effective diagnostic methods [60]. 

## Sesame 

Amongst the different oilseeds (Table 5), sesame (*Sesamum indicum*) is more and more recognized as an important food allergen source. In 2014, the sensitization rate in Europe was 4.5% [69]. The U.S. Food Allergy Safety, Treatment, Education, and Research Act of 2021 (FASTER Act) (Public Law 117-11) recommended to add sesame as a major food allergen to the list of the “Big 8” food allergens (https://www.fda.gov/regulatory-information/search-fda-guidance-documents/guidance-fda-staff-and-interested-parties-evaluating-public-health-importance-food-allergens-other, January 2026). Sesame is an important example for the quite understudied oilseed allergen sources: Although allergens from various allergen families have already been described for sesame, most of them – including oleosins – are not yet available for routine diagnostics. Therefore, in vitro diagnostics often cannot confirm the medical history as presented in the case report below. 

### Case report 

A 51-year-old male patient without known prior allergies had experienced three allergic reactions after consumption of sesame-containing meals [70 (abstract only)]: On two incidences he had consumed bread rolls sprinkled with sesame seeds for breakfast. Another incidence involved the consumption of an Asian salad containing sesame seeds. The following symptoms developed after 30 minutes of consumption, which all resolved spontaneously without anti-allergic treatment: The first time he had a flush and a light thoracic pressure, whereas the other two events were predominated by generalized urticaria. Although a sesame allergy could be assumed, SPTs were negative for the food he had consumed including sesame. On the other hand, positive SPTs to apple, sunflower seeds, and peanut were not clinically relevant. 

Both total serum IgE (11.6 IU/mL; < 100 IU/mL) and serum tryptase (5.5 µg/L; < 11.4 µg/L) were within the normal range. IgE concentrations as determined by Immuno-capacity (ImmunoCAP) (Thermo Fisher Scientific, Uppsala, Sweden) for sesame, wheat flour, rTri a 19 (wheat ω5-gliadin), rTri a 14 (wheat LTP), apple, green apple, rMal d 1 (apple PR-10, Bet v 1 homologue), rMal d 3 (apple LTP), peanut, Brazil nut, and lupine seed were negative. The patient was also IgE negative for the more recently commercially available sesame component Ses i 1 (storage protein) (Thermo Fisher Scientific, Uppsala, Sweden) (Table 5). 

**Figure 1.**
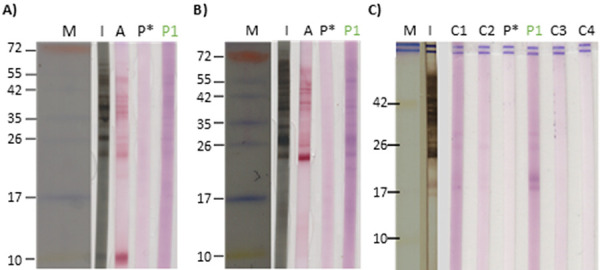
A, B, C: Immunoblot with A) an acidic sesame extract, and with B) basic sesame extract: M = marker (kDa); I = India ink; A = Auro Dye; P* = non-allergic individual; P1 = sesame-allergic patient. C) Immunoblot with peanut oleosin-enriched extract from roasted sesame seed. M = marker (kDa); I = India ink; C1 = anti-Ara h 14 antibody; C2 = anti-Ara h 15 antibody; P* = non-allergic individual; P1 = sesame-allergic patient; C3 = anti-rabbit IgG-AP, C4 = anti-human IgE-AP.

The exasperated patient performed an unauthorized oral challenge at home with a bread roll containing sesame, and developed generalized urticaria 30 minutes later. He wanted to confirm his own result at the Research Center Borstel by experimental investigations in the research group of Clinical and Molecular Allergology. An immunoblot was performed using both acidic and basic sesame seed extracts (Figure 1), where various IgE-reactive bands between 20 and 70 kDa were detected, especially in the immunoblot using the basic sesame extract. The subsequent mass spectrometry analysis identified IgE-binding seed storage proteins. 

Similar results were obtained by a small study where routine diagnostic IgE and SPTs failed to confirm sesame allergy in 9/10 adult patients with a positive – and in some cases severe – OFC to sesame [71]. These cases demonstrate that routine diagnostics by sIgE detection and SPTs often fail to confirm sesame allergy, as important allergens seem to be missing within the commercially available sesame extracts. Hence, there is an urgent need for integrating more sesame allergens into allergy diagnostic tests. 

Consequently, such cases require a workup in the laboratory, and/or physicians can use the prick-to-prick test, for which patients should bring along the suspected food components that they had consumed prior to the reaction. For sesame allergy, differences in allergenicity based on sesame processing should be kept in mind: Baked intact sesame seeds may be less allergenic and should be evaluated separately from sesame paste [72]. In those cases, the prick-to-prick test with tahini, a paste made from peeled, roasted, and finely ground sesame seeds, is recommended as a more reliable diagnostic tool [8]. Tahini is primarily used in halva, hummus, and more recently in various dips and sauces. 

## Wheat 

Wheat (*Triticum aestivum*) is an important food source worldwide. It is also used in non-food-related products such as cosmetics. However, wheat can also cause diseases including autoimmune disorders (e.g., celiac disease) and allergies. Three types of sensitization routes can be discriminated: Inhaled wheat flour can trigger baker’s asthma, which is one of the leading causes of occupational diseases (reviewed by [73]). Hydrolyzed wheat protein in cosmetics can cause skin reactions, but these patients may also develop a severe co-factor-dependent food allergy [74]. Lastly, wheat can cause food allergy and is amongst the “Big 9” food allergen sources. 

Wheat proteins can be discriminated by their solubility (Osborne fractions): hydrophilic albumins and globulins account for 15 – 20% of the wheat flour proteins, whereas the water-insoluble gluten (gliadins and glutenins) make up the majority of wheat flour proteins with 80 – 85% [75]. Due to their hydrophobic properties, gluten proteins are underrepresented in aqueous IgE or SPT testing solutions [76]. Wheat flour extract, gliadins, gluten, naturally purified α-amylase/trypsin inhibitors (nTri a aA_TI), and the recombinantly expressed wheat components non-specific LTP (Tri a 14) and ω5-gliadin (Tri a 19) are commercially available. To date, the WHO/IUIS Allergen Nomenclature Subcommittee lists 28 wheat allergens (www.allergen.org, January 2026). Many of them are relevant in both respiratory allergy and food allergy [76] (Table 6). In addition, patients often have a heterogenous IgE reaction towards these allergens. Therefore, it is crucial to elucidate the medical history and – in cases of food allergy – to have the patient’s dietary journal. Guidelines for wheat allergy diagnostics have been published by Dramburg et al. [8] and Worm et al. [77], but here, we focus on the in vitro and ex vivo diagnostics: 

Wheat food allergy is quite common in children, however, between 65 and 96% of them develop tolerance when reaching adolescence [78, 79, 80]. A high IgE concentration to wheat extract (≥ 50 kU/L) is an indicator for wheat allergy that persists into adulthood [78, 80]. Some studies showed that Tri a 19 sensitization in children was a risk factor for severe allergic reactions including anaphylaxis [81, 82, 83]. Interestingly, the sensitization profile differs between children and adults [84]. Adults are also more prone to wheat-dependent anaphylactic reactions than children [5]. The point prevalence of wheat allergy in European adults is 0.5%, which differs between countries [6, 69]. Wheat extract is commonly used in routine IgE diagnostics but has a low specificity due to cross-sensitizations with grass pollen [18] and/or CCDs [85]. A recently published meta-analysis [10] highlighted that wheat extract had a sensitivity of 72% and specificity of 79%, whilst Tri a 19 had 79% and 78%, respectively. A Tri a 19 sensitization can be detected in ≥ 79% of patients with wheat-dependent exercise-induced anaphylaxis (WDEIA), and is therefore widely accepted as marker allergen for this condition [86, 87, 88, 89, 90]. In contrast to the immediate-type wheat allergy, where symptoms occur 1 – 2 hours after exposure, WDEIA patients can experience severe symptoms several hours (2 – 4 hours) after wheat consumption. The condition is usually dependent on one or more co-factors, above all exercise, followed by nonsteroidal anti-inflammatory drugs (NSAIDs) and alcohol [90, 91, 92]. WDEIA only affects a small number of adults (0.1 – 0.8%) [93], and it occurs more frequently in Northern Europe than Southern Europe [90]. Other proteins associated with WDEIA are glutenins and other gliadins [92], but also α-amylase/trypsin inhibitors and Tri a 14 [94, 95, 96, 97], which are usually associated with baker’s asthma [76]. A few studies suggested to determine IgE levels from both high-molecular-weight (HMW) glutenin and Tri a 19 to improve the diagnosis of WDEIA [98, 99], however, only gluten extract is commercially available. 

Interestingly, only around 30% of people sensitized to Tri a 14 had a wheat food allergy [86, 100], and co-factors were required for some of them [86]. A Tri a 14-sensitization may also be based on cross-sensitization with grass pollen [76], or it can be a marker for the LTP syndrome [17]. Other potential biomarkers for wheat food allergy are the LTP from *Triticum turgidum *ssp*. durum* (Tri tu 14) [101] and α-purothionin from bread wheat (Tri a 37). Patients sensitized to Tri a 37 had a more than 4-fold increased risk of anaphylaxis [102, 103]. 

Traditionally, wheat allergy diagnostics is based on in vitro sIgE detection and/or OFC. The ex vivo BAT is only used in some cases where the routine in vitro diagnostics did not detect sufficient levels of IgE [104, 105]. A few studies have used BAT for wheat allergy diagnosis [95, 97, 106, 107, 108, 109], showing for example that it can be used to discriminate between food allergy and tolerance [109]. Similar to the sIgE data [98, 99], Gabler et al. [97, 107] also demonstrated that a combination of HMW glutenin and Tri a 19 yielded the best sensitivity and specificity using BAT. It remains to be elucidated whether the BAT will substitute the dangerous food challenge tests in the future, which are still the gold standard in wheat allergy diagnostics. 

Patients with wheat allergy should avoid food products containing wheat. Patients suffering from WDEIA need to avoid exercise (and/or other co-factors) 4 – 6 hours after a wheat-based meal [92]. As the recurrence of anaphylactic reactions is the highest for wheat-allergic patients (70.1%) [5], it is recommended that patients at risk of anaphylaxis carry an epinephrine auto-injector. 

## Tree nuts 

Besides legumes, many tree nuts are among the preferred ingredients for vegetarian or vegan diets. At the same time, they are highly potent allergen sources (Table 7), and are therefore listed among the “Big 9” food allergens. 

Sensitization to hazelnut (*Corylus avellana*) occurs in 8.1% of European adults, though the lifetime prevalence of self-reported physician-diagnosed hazelnut allergy is much lower with 0.8% [110]. Hazelnuts are a popular ingredient in snacks and sweets often consumed by children. Children are particularly affected by allergic reactions, some of which can be severe [5]. Several allergens that have been implicated in anaphylaxis are available in routine diagnostics, including the storage proteins Cor a 9 and 14, and the LTP Cor a 8 (Table 7). The storage protein family of 2S albumins is present in different tree nuts (e.g., Cor a 14, Ana o 3 (cashew), Jug r 1 (walnut)) and sesame (Ses i 1), and frequently associated with anaphylaxis [8]. Other triggers of severe reactions, namely oleosins, have been described for tree nuts including hazelnut and seeds (e.g., sesame), but are not yet available for diagnostics [14]. 

Cashews (*Anacardium occidentale*) are mainly consumed in their roasted form, but they are also used as an ingredient in processed food such as pesto, pastries, and confectionery. Not many European studies indicate the prevalence of cashew allergy, which is currently at 0.8% based on a recent review and meta-analysis [110]. An IgE titer of 2.0 kU/L to the cashew component Ana o 3 was highly predictive of a clinically relevant cashew allergy in children [111]. In silico analysis also revealed the presence of Bet v 1 (PR-10) homologues in cashew, which can potentially trigger a pollen-associated food allergy [112]. Two-thirds of patients with walnut (*Juglans regia*) and cashew allergy also had an allergic reaction to pistachio (*Pistacia vera*) and pecan (*Carya illinoinensis*), respectively. Vice versa, all of the patients with pistachio and pecan allergy experienced allergic reactions to walnut and cashew, respectively [113]. 

Two new vicilin-like allergens from hazelnut (Cor a 16) [114] and walnut (Jug r 6) [115] will be available by Thermo Fisher Scientific soon (Table 7). The prevalence of Cor a 16 sensitization was 17% in a study with 106 hazelnut-allergic patients [114]. In comparison, 26% of patients with walnut allergy (n = 77) reacted to Jug r 6, which was also shown to cause IgE cross-reactions with homologues present in hazelnut, sesame, and pistachio [115]. The new commercial components could help to elucidate associations of these allergens with clinical symptoms. 

## Fruits 

Similar to vegetable allergy, allergic reactions towards fruits (Tables 8, Table 9) can either derive from cross-reactions with pollen or from a primary sensitization (reviewed by [116]). About 10% of the population is affected by fruit allergy worldwide, though the prevalence differs between geographic regions [116, 117, 118]. The most common fruits associated with food allergies include apple (*Malus domestica*), peach (*Prunus persica*), banana (*Musa acuminata*), and kiwi (*Actinidia deliciosa*) [117, 118]. Here, we will focus on the importance of the protein families of LTPs, GRPs, and thaumatin-like proteins in the detection of fruit allergy. Interestingly, LTP proteins are predominantly found in the fruit’s peel, whereas GRPs are predominantly located in the pulp [119]. In addition, the quantities of LTP or GRPs vary between different cultivars and/or ripeness stages [119]. 

### Lipid transfer proteins 

LTPs are small proteins around 9 kDa and are resistant to heat and digestion. Systemic allergic reactions to LTPs are usually preceded by an oral allergy syndrome (OAS). For a long time, LTP was considered as a primary sensitizer in the Mediterranean region, mostly induced by peach consumption. There, it is often associated with severe allergic reactions [8]. However, over the years, more and more reports and publications indicate that LTPs also cause severe allergic reactions in Northern Europeans [64, 120, 121], especially in cases where there is no Bet v 1 (PR-10) sensitization as presented in the case report below. 

### Case report 

A 40-year-old female without a prior history of allergy suffered from three episodes of allergic reactions after food consumption in connection with exercise (for details see [120]). Symptoms included facial angioedema of the lips and tongue, sweating, an itchy scalp, generalized urticaria, dizziness, and dyspnea. Prick-to-prick testing revealed a strong reaction towards all apple components which did not seem to be clinically relevant at the time due to the absence of allergic rhinitis or OAS. 

Both total IgE (67.1 kU/L) and serum tryptase were within the normal range. In vitro allergy diagnostics by ImmunoCAP revealed no allergen-specific IgE sensitization towards Bet v 1 or to extracts of the consumed food components apart from apples (2.8 kU/L). However, a borderline positive sIgE concentration was detected for wheat LTP rTri a 14 (0.36 kU/L). Subsequently, other LTPs from different allergen sources were tested in vitro, revealing a sensitization towards LTP proteins from different sources, namely apple Mal d 3 (13.10 kU/L), peach Pru p 3 (6.25 kU/L), peanut Ara h 9 (0.67 kU/L), and hazelnut Cor a 8 (0.53 kU/L). 

In conclusion, the patient’s sensitization towards apples did not derive from an IgE-reaction to the Bet v 1 homologue Mal d 1, but rather from the strong sensitization towards the apple LTP Mal d 3. Given the medical history and the detected IgE reactivity towards apple LTP and multiple other LTPs, a food-dependent exercise-induced anaphylaxis (FDEIA) triggered by LTP sensitization and exercise was diagnosed [120]. The patient was advised to avoid consuming fruits of the *Rosaceae* family (peach, apple, apricot, plum, cherry, and pear), particularly in association with exercise (Table 8). An adrenaline auto-injector, oral cetirizine, and prednisolone were prescribed, and an anaphylaxis action plan was provided and explained. The severity of the LTP syndrome is associated with the number of LTPs to which a patient is sensitized [64]. Interestingly, patients seem to develop less severe reactions in cases of co-sensitizations to Bet v 1 and profilin homologues [122]. This case report highlights the importance of determining the patient’s sensitization profile to make a diagnosis. 

### Gibberellin-regulated proteins (GRPs) 

GRPs are small, non-glycosylated, monomeric, heat- and digestion-resistant proteins with antimicrobial activity (reviewed by [16]). They can be found in tree pollen (e.g., *Cupressaceae*) and plant-based food such as peaches, citrus fruits, apricots, cherries, and pomegranates. Hence, both primary and secondary pollen-associated sensitization to GRPs can occur [116]. GRPs can trigger severe systemic reactions with and without the presence of co-factors [16]. A GRP-based allergy was first described in 2013 in patients with severe reactions to peaches [123]. The patients were monosensitized to peach GRP, which was officially registered as Pru p 7 by the WHO/IUIS Allergen Nomenclature Subcommittee. Several other GRPs have been identified as clinically relevant fruit allergens (e.g., Pru m 7 (Japanese apricot), Cit s 7 (orange)) (Table 8), which can trigger IgE cross-reactions [16]. Despite their significance in anaphylactic reactions, GRP molecules have not been implemented in routine diagnostics yet apart from Pru p 7. 

### Thaumatin-like proteins 

Similar to GRPs, thaumatin-like proteins (PR-5) (reviewed by [124]) also play a role in plant defense and occur in pollen and fruits. Hence, they may cause primary or secondary (pollen-associated) food allergy. They are present in different fruits such as apples, peaches, bananas or kiwis (Table 8). Allergic symptoms to thaumatin-like proteins can range between OAS and anaphylaxis [124]. Their structure suggests a high resistance to food processing by heat or enzymes [125]. 

### Case report 

A 29-year-old woman experienced anaphylactic reactions (serum tryptase: 2.9 µg/L; total serum IgE: 16.3 kU/L) to both banana (*Musa acuminata*) and kiwi (*Actinidia deliciosa*) at two separate events (for details see [126]). This was indicative of a cross-reaction between both fruits, however, IgE tests were negative for bananas. Together with a team in Belgrade (Serbia), who had purified different banana allergens, our group could prove that the patient suffered from an IgE-mediated allergy to Mus a 2 (chitinase), Mus a 4 (thaumatin-like protein), and Mus a 5 (β-1,3-glucanase). In addition, our group was able to detect an IgE cross-reactivity to kiwi mediated by the thaumatin-like protein (Mus a 4 from banana and Act d 2 from kiwi fruit). An IgE reactivity to Act d 1 and whole kiwi extract was also detected, which explains the allergic reaction first to kiwi fruit and the subsequent reaction to banana, although a sensitization to whole banana extract was not observed [126]. Kiwi allergy is one of the most frequent fruit allergies in Europe and other countries [116]. Whilst different components for kiwi are already available for routine diagnostics, none of the banana allergens are provided in the current test panels. Our case report is a striking example of the fact that IgE diagnostics for fruits still lacks relevant components to assess sensitization and IgE cross-reactivity. 

## Future perspectives 

To date, diagnostics for plant allergies is still reliant on protein extracts. However, these tests are accompanied by low sensitivity and specificity as cross-sensitizations between plants are frequent, particularly with regard to pollen-associated food allergy. Wherever they are commercially available or published, individual allergens are already improving the sensitivity and specificity of in vitro and ex vivo allergy diagnostics. Some allergens show biomarker potential for certain allergies and can even indicate the severity of an allergic reaction including anaphylaxis. Determining the “theratype” by molecular allergology will help to decide which therapeutic options will benefit a patient the most. However, several clinically relevant allergens are not included in routine diagnostic panels yet, which is partly due to difficulties in purification or production procedures required (e.g. for oleosins). 

The currently available in vitro IgE tests are sometimes complemented by ex vivo tests (e.g., BAT), which showed promising results in differential diagnostics, e.g., of peanut and wheat allergies. It remains to be elucidated whether the BAT might – at least partly – replace the dangerous OFCs. An improved BAT could very well be available on the market in the next 5 – 10 years. The BAT could help to monitor the course as well as the success of therapeutic interventions. In addition, it might help to examine potential cross-reactions vs. cross-sensitizations with other food sources. 

One of the remaining challenges in diagnostics is to determine allergens and assess allergenic activity of highly processed food, which has become a part of daily life for many, including those consuming meat-substitution products. Some studies have already shown that food processing can have an impact on the allergenicity in both ways, which in turn can affect allergy symptoms (reviewed by [60, 127]). Therefore, a more comprehensive labeling on these products including more – and potentially cross-reacting – allergen sources as well as indicating the amount of allergen present might benefit people with specific food allergies. Repeated attempts to “cut out” responsible single allergens from allergen sources has not or not entirely been successful as this often affects plant growth or taste. 

Different nutrients have been and are still integrated into the European market due to the effects of globalization, increased health awareness, and concerns over planetary health. Therefore, it is important to keep track of emerging allergies and the allergenic components responsible to improve patient care. Understanding the phylogenetic relationship between plants has been advantageous to predict cross-sensitizations but should not replace the need for marker allergens to simplify the diagnosis. 

## Authors’ contributions 

EMR: First author, main editing and main revision of the manuscript, management of references, contributing author of the wheat section, revised abstract, revised introduction, and future perspectives. MP: Contributing author of the peanut section, text editing and revision of the manuscript. MAR: Contributing author of the legume section, creation of the tables. UJ: Corresponding author, contributing author of all the other sections not mentioned above, future perspectives, creation of Figure 1, editing and revision of the manuscript. 

## Funding 

UJ: Projects are funded by the BMFTR (FKZ 82DZL001A1; FKZ 82DZL001C1), and DFG (JA 1007/2-1; JA 1007/2-3; JA 1007/4-1). MAR is funded by the BMFTR (DZL: FKZ 82DZL001C1). EMR was funded by the BMFTR INDICATE-FH (FKZ: 01EA2109B) and is presently funded by the RCB. However, for the writing of the manuscript no funding was obtained. 

## Conflict of interest 

UJ: UJ has received two hotel accommodations and catering for both a lecture and the chairing of a workshop/symposium organized by ALK-Abelló (Hamburg, Germany). One respective honorarium went to her institution, the Research Center Borstel (RCB). The honorarium for a lecture invited and sponsored by Thermo Fisher Diagnostik Austria also went to her institution, the RCB. Her research on molecular allergology is funded by the Federal Ministry of Research, Technology and Space (BMFTR) (DZL (FKZ 82DZL001C1) and INDICATE-FH (01EA2109B)), the Federal Ministries of Technology, Economy and Technology, Food and Agriculture (BMEL), the German Research Foundation (DFG) (JA 1007/2-1; JA 1007/2-3; JA 1007/4-1), and the Kanert Foundation. EMR: Her position has been funded by the BMFTR (INDICATE-FH, 01EA2109B) for three years for the project on wheat hypersensitivity. MAR: Her position is currently funded by the DZL FKZ 82DZL001C1). MP declares no conflicts of interest. 

**Table 1.** Known food allergens from vegetables listed by the WHO/IUIS allergen database (www.allergen.org, January 2026).

**Source**	**Allergen**	**MW (kDa)**	**Functional category**
*Apium graveolens* (Celery)	Api g 1^a^	16	Pathogenesis-related protein, PR-10, Bet v 1 family member
	Api g 2	9	Non-specific lipid transfer protein type 1 (nsLTP1)
	Api g 3	28	Chlorophyll a-b binding protein, chloroplast
	Api g 4	14	Profilin
	Api g 5	58	FAD-containing oxidase
	Api g 6	7	Non-specific lipid transfer protein type 2 (nsLTP2)
	Api g 7	12	Defensin like protein 1
*Asparagus officinalis *(Asparagus)	Aspa o 1	9	Non-specific lipid transfer protein type 1 (nsLTP1)
*Brassica oleracea* (Cabbage and others)	Bra o 3	9	Non-specific lipid transfer protein type 1 (nsLTP1)
*Daucus carota* (Carrot)	Dau c 1^b^	16	Pathogenesis-related protein, PR-10, Bet v 1 family member
	Dau c 4	14	Profilin
	Dau c 5	33	Isoflavone reductase-like protein
*Lactuca sativa* (Cultivated lettuce)	Lac s 1	9	Non-specific lipid transfer protein type 1 (nsLTP1)
*Manihot esculenta* (Cassava, manioc)	Man e 5	30	Glutamic acid-rich protein
*Capsicum annuum* (Chili, bell pepper)	Cap a 1	23	Osmotin-like protein (thaumatin-like protein)
	Cap a 2	14	Profilin
	Cap a 7	12	Gibberellin-regulated protein, snakin
*Cucurbita maxima *(Pumpkin)	Cuc ma 4	50	11S globulin
	Cuc ma 5	14	2S albumin
*Solanum melongena *(Eggplant)	Sola m 1	17	Profilin
*Solanum tuberosum* (Potato)	Sola t 1	43	Patatin
	Sola t 2	21	Cathepsin D inhibitor PDI, aspartic protease inhibitor 11, Kunitz-type protease inhibitor
	Sola t 3	21	Cysteine protease inhibitor 1, cysteine protease inhibitor 10, Kunitz-type protease inhibitor
	Sola t 4	16	Serine protease inhibitor 7, Kunitz-type protease inhibitor, PIG
*Solanum lycopersicum* (Tomato)	Sola l 1	14	Profilin
	Sola l 2	50	β-fructofuranosidase
	Sola l 3	9	Non-specific lipid transfer protein type 1 (nsLTP1)
	Sola l 4	20	Pathogenesis-related protein, PR-10, Bet v 1 family member, TSI-1
	Sola l 5	19	Cyclophilin
	Sola l 6	7	Non-specific lipid transfer protein type 2 (nsLTP2)
	Sola l 7	12.5	Non-specific lipid transfer protein type 1 (nsLTP1)

IgE diagnostics available from ^a^Thermo Fisher Scientific (Uppsala, Sweden), ^b^Dr Fooke (Neuss, Germany).

**Table 2.** Known food allergens from medicinal root/spices listed by the WHO/IUIS allergen database (www.allergen.org, January 2026).

**Source**	**Allergen**	**MW (kDa)**	**Functional category**
*Panax ginseng* (Korean ginseng)	Pana g 1	17	Pathogenesis-related protein PR-10-1, Bet v 1-related protein, ribonuclease 1
*Zanthoxylum bungeanum* (Sichuan pepper)	Zan b 1	14	2S albumin
	Zan b 2	50	11S globulin, legumin

**Table 3.** Known food allergens from peanut listed by the WHO/IUIS allergen database (www.allergen.org, January 2026).

**Source**	**Allergen**	**MW (kDa)**	**Functional category**
*Arachis hypogaea* (Peanut)	Ara h 1^a,b^	64	Vicilin, 7S globulin seed storage protein
	Ara h 2^a,b,c,d^	17	Conglutin (2S albumin)
	Ara h 3^a,b^	60	Legumin-like protein
	Ara h 5	15	Profilin
	Ara h 6^a,b^	15	Conglutin (2S albumin)
	Ara h 7	15	Conglutin (2S albumin)
	Ara h 8^a^	17	Pathogenesis-related protein, PR-10, Bet v 1 family member
	Ara h 9^a,b^	9.8	Non-specific lipid transfer protein type 1 (nsLTP1)
	Ara h 10	16	Oleosin
	Ara h 11	14	Oleosin
	Ara h 12	< 12	Defensin
	Ara h 13	< 11	Defensin
	Ara h 14	17.5	Oleosin
	Ara h 15	17	Oleosin
	Ara h 16	8.5	Non-specific lipid transfer protein type 2 (nsLTP2)
	Ara h 17	11	Non-specific lipid transfer protein type 2 (nsLTP2)
	Ara h 18	21	Cyclophilin, peptidyl-prolyl cis-trans isomerase
	Ara h 19	39	Annexin Gh1
	Ara h 20	57	Seed biotinylated protein SBP65

IgE diagnostics available from ^a^Thermo Fisher Scientific (Uppsala, Sweden), ^b^Dr Fooke (Neuss, Germany), ^c^Euroimmun (Lübeck, Germany), ^d^Gold Standard Diagnostics (Kassel, Germany).

**Table 4.** Known food allergens from legumes (except peanuts) listed by the WHO/IUIS allergen database (www.allergen.org, January 2026).

**Source**	**Allergen**	**MW (kDa)**	**Functional category**
*Cicer arietinum* (Chickpea)	Cic a 1	42	Late embryogenesis abundant protein family 4
*Glycine max* (Soybean)	Gly m 1	7	Hydrophobic protein
	Gly m 2	8	Defensin
	Gly m 3	14	Profilin
	Gly m 4^a^	17	Pathogenesis-related protein, PR-10, Bet v 1 family member
	Gly m 5^a^	48	β-conglycinin (vicilin, 7S globulin)
	Gly m 6^a^	55	Glycinin (legumin, 11S globulin)
	Gly m 7	76.2	Seed biotinylated protein
	Gly m 8	28	2S albumin
*Lens culinaris* (Lentil)	Len c 1	47	γ-vicilin subunit
	Len c 2	66	Seed biotinylated protein; late embryogenesis abundant protein
	Len c 3	9	Non-specific lipid transfer protein type 1 (nsLTP1)
*Lupinus albus* (White lupine)	Lup a 5	15	Profilin
*Lupinus angustifolius* (Blue lupine)	Lup an 1	55 – 61	Conglutin β (7S seed storage globulin, vicilin)
	Lup an 3	11	Non-specific lipid transfer protein type 1 (nsLTP1)
*Phaseolus vulgaris* (Kidney bean)	Pha v 3	8.8 – 9	Non-specific lipid transfer protein type 1 (nsLTP1)
*Pisum sativum* (Pea)	Pis s 1	44	Vicilin
	Pis s 2	63	Convicilin
	Pis s 3	9.5	Non-specific lipid transfer protein type 1 (nsLTP1)
*Vigna radiata* (Mung bean)	Vig r 1	16	Pathogenesis-related protein, PR-10, Bet v 1 family member
	Vig r 2	52	8S globulin
	Vig r 3	50	renamed to Vig r 2.0201
	Vig r 4	30	Seed albumin
	Vig r 5	15	fragment of Vig r 2
	Vig r 6	18	Cytokinin-specific binding protein (CSBP), Bet v 1 family member

IgE diagnostics available from ^a^Thermo Fisher Scientific (Uppsala, Sweden).

**Table 5.** Known food allergens from oilseed crops listed by the WHO/IUIS allergen database (www.allergen.org, January 2026).

**Source**	**Allergen**	**MW (kDa)**	**Functional category**
*Brassica juncea* (Indian or oriental mustard)	Bra j 1	14	2S albumin
*Brassica napus* (Rapeseed)	Bra n 1	15	2S albumin, napin BnIII, napin-3
*Brassica rapa* (Field mustard, turnip)	Bra r 1	10 – 14	2S albumin, napins
	Bra r 2	25	Prohevein homologue
*Helianthus annuus* (Sunflower)	Hel a 2	14.7	Profilin
	Hel a 3	9	Non-specific lipid transfer protein type 1 (nsLTP1)
	Hel a 15	14	2S albumin
	Hel a 16	15	2S albumin
	Hel a 17	10	2S albumin
*Linum usitatissimum* (Flaxseed)	Lin u 1	10 – 13	2S albumin, conlinin
*Papaver somniferum* (Opium poppy)	Pap s 1	10 – 50	Vicilin (7S globulin) with N-terminal α-hairpinin peptides
	Pap s 2	52	Legumin (11S globulin)
	Pap s 3	10	Late embryogenesis abundant protein 5 (LEA-5); small hydrophilic seed protein
*Ricinus communis* (Castor bean)	Ric c 1	11	2S albumin
*Sesamum indicum* (Sesame)	Ses i 1^a^	9	2S albumin
	Ses i 2	7	2S albumin
	Ses i 3	45	7S vicilin-like globulin
	Ses i 4	17	Oleosin
	Ses i 5	15	Oleosin
	Ses i 6	52	11S globulin
	Ses i 7	57	11S globulin
*Sinapis alba* (Yellow mustard)	Sin a 1	14	2S albumin
	Sin a 2	51	11S globulin, legumin-like
	Sin a 3	12.3	Non-specific lipid transfer protein type 1 (nsLTP1)
	Sin a 4	13 – 14	Profilin

IgE diagnostics available from ^a^Thermo Fisher Scientific (Uppsala, Sweden).

**Table 6.** Known food allergens from cultivated grain crops listed by the WHO/IUIS allergen database (www.allergen.org, January 2026).

**Source**	**Allergen**	**MW (kDa)**	**Functional category**
*Fagopyrum esculentum* (Common buckwheat)	Fag e 2^a^	16	2S albumin
	Fag e 3	19	α-hairpinin
	Fag e 4	3.9	Hevein-like antimicrobial peptide
	Fag e 5	55	8S globulin, vicilin-like
*Fagopyrum tataricum* (Tartarian buckwheat)	Fag t 2	16	2S albumin
	Fag t 6	18	Oleosin
*Hordeum vulgare* (Barley)	Hor v 12	14	Profilin
	Hor v 15	14.5	Monomeric α-amylase inhibitor BMAI-1
	Hor v 16	47.8	α-amylase
	Hor v 17	57.3	β-amylase
	Hor v 20	34	γ-hordein 3
*Secale cereale* (Rye)	Sec c 20	70	γ-secalin, coeliac immunoreactive protein
*Triticum aestivum* (Wheat)	Tri a 12	14	Profilin
	Tri a 14^a^	9	Non-specific lipid-transfer protein type 1 (nsLTP1)
	Tri a 17	56	β-amylase
	Tri a 18	21	Agglutinin isolectin 1, isolectin A, wheat germ agglutinin 1 (WGA1)
	Tri a 19^a^	65	ω5-gliadin
	Tri a 20	35 – 38	γ-gliadin
	Tri a 21		α/β-gliadin
	Tri a 26	88	High molecular weight glutenin
	Tri a 29	13	α-amylase inhibitor (tetramer) CM1/CM2
	Tri a 30	16	α-amylase inhibitor (tetramer) CM3
	Tri a 33		Serin protease inhibitor (Serpin)
	Tri a 36	40	Low molecular weight glutenin
	Tri a 37	12	α-purothionin
	Tri a 40	15.96	α-amylase inhibitor (tetramer) CM17
*Triticum turgidum ssp durum* (Durum wheat)	Tri tu 14	9.2	Non-specific lipid transfer protein 1 (nsLTP1)
*Zea mays* (Maize)	Zea m 8	28.6	Class IV chitinase
	Zea m 14	9	Non-specific lipid transfer protein type 1 (nsLTP1)

IgE diagnostics available from ^a^ Thermo Fisher Scientific (Uppsala, Sweden).

**Table 7.** Known food allergens from tree nuts listed by the WHO/IUIS allergen database (www.allergen.org, Jan 2026).

**Source**	**Allergen**	**MW (kDa)**	**Functional category**
*Anacardium occidentale* (Cashew)	Ana o 1	50	Vicilin-like protein
	Ana o 2^a^	55	Legumin-like protein
	Ana o 3^a^	14	2S albumin
*Bertholletia excelsa* (Brazil nut)	Ber e 1^a^	9	2S sulfur-rich seed storage albumin
	Ber e 2	29	11S globulin
*Carya illinoinensis* (Pecan)	Car i 1	16	2S albumin
	Car i 2	55	Vicilin-like seed storage protein; 7S globulin
	Car i 4	55	11S globulin, legumin
*Castanea sativa* (Chestnut)	Cas s 5	16	Chitinase class I
	Cas s 8	55	Non-specific lipid transfer protein type 1 (nsLTP1)
	Cas s 9	17	Cytosolic class I small heat shock protein; Hsp20
*Corylus avellana* (Hazelnut)	Cor a 2	14	Profilin
	Cor a 8^a,b^	9	Non-specific lipid transfer protein type 1 (nsLTP1)
	Cor a 9^a,b^	40	11S globulin (legumin-like)
	Cor a 11	48	7S seed storage globulin (vicilin-like)
	Cor a 12	17	Oleosin
	Cor a 13	14 – 16	Oleosin
	Cor a 14^a^	10	2S albumin
	Cor a 15	17	Oleosin
	Cor a 16^a^	6 – 8, 48	7S globulin, vicilin-like
*Juglans nigra* (Black walnut)	Jug n 1	15	2S albumin
	Jug n 2	56	Vicilin (7S globulin)
	Jug n 4	22 – 34	11S globulin (legumin-like)
*Juglans regia* (English walnut)	Jug r 1^a^	15 – 16	2S albumin
	Jug r 2	44	7S globulin
	Jug r 3^a^	9	Non-specific lipid transfer protein type 1 (nsLTP1)
	Jug r 4	58.1	11S globulin
	Jug r 5	20	PR-10 protein
	Jug r 6^a^	47	7S globulin, vicilin
	Jug r 7	13	Profilin
	Jug r 8	9	Non-specific lipid transfer protein type 2 (nsLTP2)
	Jug r 9	92	Phospholipase D α 1
*Macadamia integrifolia* (Macadamia)	Mac i 1	50	Vicilin
	Mac i 2	60	Legumin
*Pinus koraiensis* (Korean pine)	Pin k 2	48	Vicilin
*Pinus pinea* (Stone pine)	Pin p 1	15	2S albumin
*Pistacia vera* (Pistachio)	Pis v 1	7	2S albumin
	Pis v 2	32	11S globulin subunit
	Pis v 3	55	Vicilin
	Pis v 4	25.7	Manganese superoxide dismutase
	Pis v 5	36 (acidic subunit)	11S globulin subunit
*Prunus dulcis* (Almond)	Pru du 1	18	PR-10, Bet v 1-related protein
	Pru du 3	9	Non-specific lipid transfer protein type 1 (nsLTP1)
	Pru du 4	14	Profilin
	Pru du 5	10	60S acidic ribosomal protein P2
	Pru du 6	360 (hexamer)	Amandin, 11S globulin, legumin-like
	Pru du 8	31	α-hairpinin
	Pru du 10	60	Mandelonitrile lyase 2; hydroxynitrile lyase

IgE diagnostics available from ^a^Thermo Fisher Scientific (Uppsala, Sweden), ^b^Dr Fooke (Neuss, Germany).

**Table 8.** Known food allergens from fruits listed by the WHO/IUIS allergen database (www.allergen.org, January 2026).

**Source**	**Allergen**	**MW (kDa)**	**Functional category**
*Actinidia chinensis* (Gold kiwi fruit)	Act c 5	28	Kiwellin
	Act c 8	17	Pathogenesis-related protein, PR-10, Bet v 1 family member
	Act c 10	10	Non-specific lipid transfer protein type 1 (nsLTP1)
*Actinidia deliciosa* (Green kiwi fruit)	Act d 1^a^	30	Cysteine protease (actinidin)
	Act d 2^a^	24	Thaumatin-like protein
	Act d 3	40	Glycoprotein with unknown function
	Act d 4	11	Phytocystatin
	Act d 5^a^	28	Kiwellin
	Act d 6	18	Pectin methylesterase inhibitor
	Act d 7	50	Pectin methylesterase
	Act d 8^a^	17	Pathogenesis-related protein, PR-10, Bet v 1 family member
	Act d 9	14	Profilin
	Act d 10	10	Non-specific lipid transfer protein type 1 (nsLTP1)
	Act d 11	17	Major latex protein/ripening-related protein (MLP/RRP), Bet v 1 family member
	Act d 12	50	Cupin, 11S globulin
	Act d 13	11	2S albumin
*Ananas comosus* (Pineapple)	Ana c 1	15	Profilin
	Ana c 2^a^	22.8	Bromelain
*Citrullus lanatus* (Watermelon)	Citr l 2	14	Profilin
*Citrus limon* (Lemon)	Cit l 3	9.6	Non-specific lipid transfer protein type 1 (nsLTP1)
*Citrus reticulata* (Tangerine)	Cit r 3	9	Non-specific lipid transfer protein type 1 (nsLTP1)
*Citrus sinensis* (Sweet orange)	Cit s 1	23	Germin-like protein
	Cit s 2	14	Profilin
	Cit s 3	9.46	Non-specific lipid transfer protein type 1 (nsLTP1)
	Cit s 7	8	Gibberellin regulated protein, peamaclein
*Cocos nucifera* (Coconut)	Coc n 1	53	Vicilin-like protein
*Cucumis melo* (Muskmelon)	Cuc m 1	67	Alkaline serine protease (cucumisin)
	Cuc m 2	14	Profilin
	Cuc m 3	17	Pathogenesis-related protein PR-1
*Malus domestica* (Apple)	Mal d 1^a,b,c,e^	17.5	Pathogenesis-related protein, PR-10, Bet v 1 family member
	Mal d 2	23	Thaumatin-like protein
	Mal d 3^a,b^	9	Non-specific lipid transfer protein type 1 (nsLTP1)
	Mal d 4^e^		Profilin
*Musa acuminata* (Banana)	Mus a 1	15	Profilin
	Mus a 2	33	Class 1 chitinase
	Mus a 3	9	Non-specific lipid transfer protein type 1 (nsLTP1)
	Mus a 4	20	Thaumatin-like protein
	Mus a 5	30	β-1,3-glucanase
	Mus a 6	27	Ascorbate peroxidase
*Persea americana* (Avocado)	Pers a 1	32	Class I chitinase
*Prunus armeniaca* (Apricot)	Pru ar 1		Pathogenesis-related protein, PR-10, Bet v 1 family member
	Pru ar 3	9	Non-specific lipid transfer protein type 1 (nsLTP1)
	Pru ar 5	17.4	Acidic Hev b 5-like protein
*Prunus avium* (Sweet cherry)	Pru av 1^e^	9	Pathogenesis-related protein, PR-10, Bet v 1 family member
	Pru av 2	23	Thaumatin-like protein
	Pru av 3^e^	10	Non-specific lipid transfer protein type 1 (nsLTP1)
	Pru av 4^e^	15	Profilin
	Pru av 7	9	Gibberellin-regulated protein
*Prunus domestica* (European plum)	Pru d 3	9	Non-specific lipid transfer protein type 1 (nsLTP1)
*Prunus persica* (Peach)	Pru p 1^a,b^	18	Pathogenesis-related protein, PR-10, Bet v 1 family member
	Pru p 2	25 – 28	Thaumatin-like protein
	Pru p 3^a,b,e^	10	Non-specific lipid transfer protein type 1 (nsLTP1)
	Pru p 4^a,b^	14	Profilin
	Pru p 7^a^		Gibberellin-regulated protein (PF02704)
*Punica granatum* (Pomegranate)	Pun g 1	9	Non-specific lipid transfer protein 1 (nsLTP1)
	Pun g 7	7	Gibberellin regulated protein, pommaclein
	Pun g 14	29	Chitinase III
*Pyrus communis* (Pear)	Pyr c 1	18	Pathogenesis-related protein, PR-10, Bet v 1 family member
	Pyr c 3	9	Nonspecific lipid transfer protein type 1 (nsLTP1)
	Pyr c 4	14	Profilin
	Pyr c 5	34	Isoflavone reductase related protein, putative phenylcoumaran benzylic ether reductase
*Vitis vinifera* (Grape)	Vit v 1	9	Non-specific lipid transfer protein type 1 (nsLTP1)
*Carica papaya* (Papaya)	Cari p 1^d^	57	Endo-polygalacturonase
	Cari p 2	28	Chymopapain; member of the papain-like cysteine protease (petidase C1) family
*Litchi chinensis* (Lychee)	Lit c 1	15	Profilin
*Mangifera indica* (Mango)	Man i 1	28	Class IV chitinase
	Man i 2	17	PR-10 protein; Bet v 1-related protein
	Man i 4	14	Profilin
*Phoenix dactylifera* (Date palm)	Pho d 2	14	Profilin
*Prunus mume* (Japanese apricot)	Pru m 7	6.9	Gibberellin-regulated protein, peamaclein
*Ziziphus mauritiana* (Chinese date)	Ziz m 1	30	Class III chitinase

IgE diagnostics available from ^a^Thermo Fisher Scientific (Uppsala, Sweden), ^b^Dr Fooke (Neuss, Germany), ^c^Euroimmun (Lübeck, Germany), ^d^Gold Standard Diagnostics (Kassel, Germany), ^e^Siemens Healthineers (Erlangen, Germany).

**Table 9.** Known food allergens from berries listed by the WHO/IUIS allergen database (www.allergen.org, January 2026).

**Source**	**Allergen**	**MW (kDa)**	**Functional category**
*Fragaria ananassa* (Strawberry)	Fra a 1^b^	18	Pathogenesis-related protein, PR-10, Bet v 1 family member
	Fra a 3^b^	9	Non-specific lipid transfer protein type 1 (nsLTP1)
	Fra a 4	13	Profilin
*Morus nigra* (Black mulberry)	Mor n 3	10	Non-specific lipid transfer protein type 1 (nsLTP1)
*Rubus idaeus* (Red raspberry)	Rub i 1	17	Pathogenesis-related protein, PR-10, Bet v 1 family member
	Rub i 3	11	Non-specific lipid transfer protein 1 (nsLTP1)

IgE diagnostics available from ^b^Dr Fooke (Neuss, Germany).
